# A large A(H3N2) influenza outbreak with a high attack rate in a drug user community in Italy, April 2022

**DOI:** 10.1017/S0950268823000055

**Published:** 2023-01-19

**Authors:** Maria Gori, Clara Fappani, Silvia Bianchi, Marta Canuti, Daniela Colzani, Paolo Ottogalli, Sarah Duehren, Elisabetta Tanzi, Antonella Amendola, Antonio Boschini

**Affiliations:** 1 Department of Health Sciences, Università degli Studi di Milano, Milan, Italy; 2 Coordinated Research Centre EpiSoMI, Università degli studi di Milano, Milan, Italy; 3 Department of Clinical Sciences and Community Health, Università degli Studi di Milano, Milan, Italy; 4 Department of Pathophysiology and Transplantation, Università degli Studi di Milano, Milan, Italy; 5 Medical Centre, San Patrignano Community, Coriano (Rimini), Italy; 6 Coordinated Research Centre for Multidisciplinary Research in Health Science (MACH), Università degli studi di Milano, Milan, Italy

**Keywords:** Community outbreaks, influenza A(H3N2), public health

## Abstract

Despite the COVID-19 pandemic, influenza remains an important issue. Especially in community settings, influenza outbreaks can be difficult to control and can result in high attack rates. In April 2022, a large A(H3N2) influenza outbreak spread in the largest Italian drug-rehabilitation community. One hundred eighty-four individuals presented influenza-like symptoms (attack rate of 26.2%); 56% previously received the influenza vaccine. Sequence analyses highlighted a genetic drift from the vaccine strain, which may have caused the observed lack of protection.

## Background

Influenza has a major impact in community settings and can result in high attack rates, causing considerable morbidity and interruptions of daily activities [1, 2]. Seasonal vaccination of residents and caregivers contributes to protect susceptible individuals and reduce the circulation of influenza viruses within a community. Nevertheless, vaccine mismatch, which results in a reduced protection, and outbreaks can still occur. In this paper, we describe an outbreak of influenza A (H3N2) that occurred in April 2022 in the largest Italian drug recovery community, San Patrignano.

## Outbreak description

An epidemiological investigation involving all residents living in the community (*N* = 702 at the time of the outbreak) was promptly initiated. A written informed consent was obtained from each individual whose data and samples were collected for the study. The study was performed by inspecting patients’ records and no questionnaires were used. The median age of the residents was 32 years (range 13–66 years), 576 (82.1%) were male and 126 (17.9%) were female. Twelve residents (1.7%) were HIV-positive. A total of 340 individuals (48.4%) had received seasonal inactivated quadrivalent influenza vaccine in November 2021 (*N* = 330) or in January 2022 (*N* = 10).

Overall, a total of 184 individuals presented influenza-like symptoms, implying an estimated attack rate of 26.2%. The age of cases ranged from 14 to 55 years (median: 30 years); 152 were male (82.6%).

**Fig. 1.**
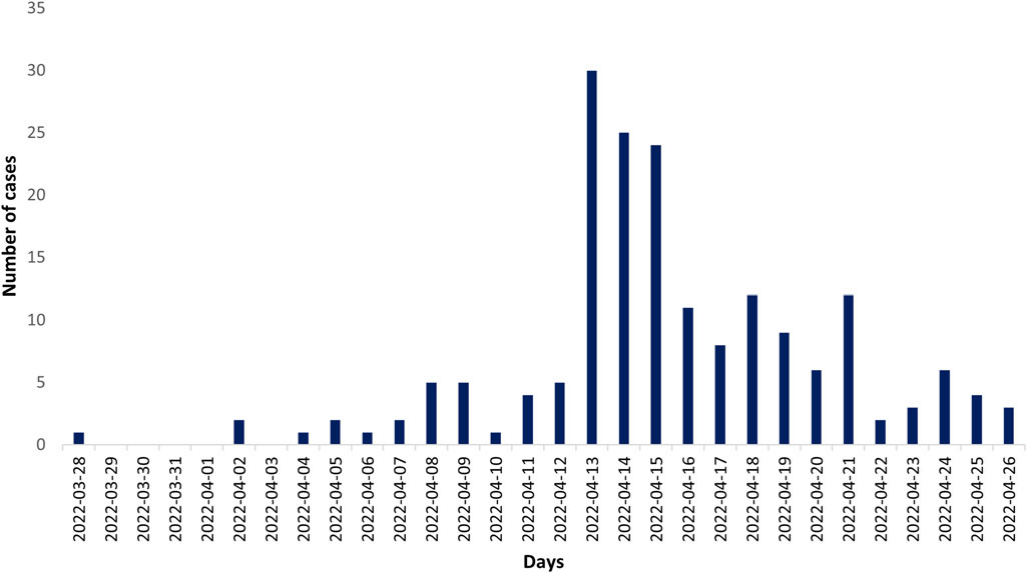
Epidemic curve of A(H3N2) influenza outbreak occurred in April 2022 in the drug-rehabilitation community San Patrignano. A case was defined as a resident with respiratory illness symptoms concurrently with fever.

The epidemic curve (Fig. 1) shows the distribution of cases based on the day of symptom onset. The first case was detected on 28 March 2022, and the peak was reached on 13 April (16 days later). The last case occurred 29 days after the first observed case.

Of the 184 residents with influenza-like symptoms, 85 (46.2%) reported fever >38.5°C and respiratory symptoms, and two individuals had gastrointestinal symptoms. Two residents experienced febrile convulsions, and three cases presented syncope. Nobody was hospitalised. Eight of the 12 HIV-infected (66.7%) and 176 of the 690 HIV-negative individuals (28.7%) developed the disease (*P*-value 0.002). HIV-positive residents had achieved immune reconstitution with HAART therapy and did not show higher fever or more severe respiratory symptoms than residents without HIV coinfection.

Out of the 184 subjects involved in the outbreak, 103 (56.0%) had received seasonal influenza vaccine, including the first detected case. Overall, 103 of the 340 (30.3%) vaccinated and 81 out of the 362 non-vaccinated individuals (22.4%) developed influenza-like symptoms (relative risk 1.35, 95% confidence interval 1.05–1.74).

San Patrignano community consists of several residential buildings, where residents live and work closely together. Residents' contacts with the outside world are limited. However, all staff (e.g. educators) live outside of the community, and residents have contacts with relatives, doctors and psychologists. A non-homogeneous distribution of cases in different working areas was observed with the highest percentage (48.7%) of positive residents working in catering and food service (Table 1, Supplementary Table S1). An attack rate significantly higher than the one calculated for the entire community (26.2%) was observed in the catering and food service (*P*-value 0.001), farm (*P*-value 0.012) and kitchen (*P*-value 0.049) areas. On the contrary, a significantly lower attack rate was found in the bakery (*P* < 0.001). After stratifying according to working area, in catering and food service, farm and kitchen area a vaccine effectiveness (VE) of 31%, 47% and 16%, respectively, was observed (Supplementary Table S1).

**Table 1.** Distribution of residents and outbreak cases among working areas in San Patrignano

Working area	Total residents (*N*)	Cases	Attack rate (%)^a^	*P* value
Animal farm	39	0	0	–
Bakery	72	5	6.9	<0.001
Blacksmith's workshop	12	3	25.0	0.482
Building	6	0	0	–
Butchery	23	8	34.8	0.176
Catering and food service	39	19	48.7	0.001
Cheese factory	57	12	21.1	0.182
Children's centre (females)	15	4	26.7	0.468
Children's centre (males)	11	3	27.3	0.451
Decoration	36	8	22.2	0.299
Plumbing and electrical maintenance	31	7	22.6	0.331
Farm	65	25	38.5	0.012
Graphic design	32	11	34.4	0.147
Kennel	22	3	13.6	0.087
Kitchen	46	17	37.0	0.049
Laundry	25	10	40.0	0.063
Medical centre	23	8	34.8	0.176
Park	74	21	28.4	0.323
Pizzeria	1	0	0	–
Vineyard	5	0	0	–
Warehouse	32	12	37.5	0.076
Weaving	36	8	22.2	0.299
Total	702	184	–	–

a The attack rate for the different settings was compared to the one calculated for the entire community (26.2%).

## Laboratory confirmation and molecular characterisation of strains

**Fig. 2.**
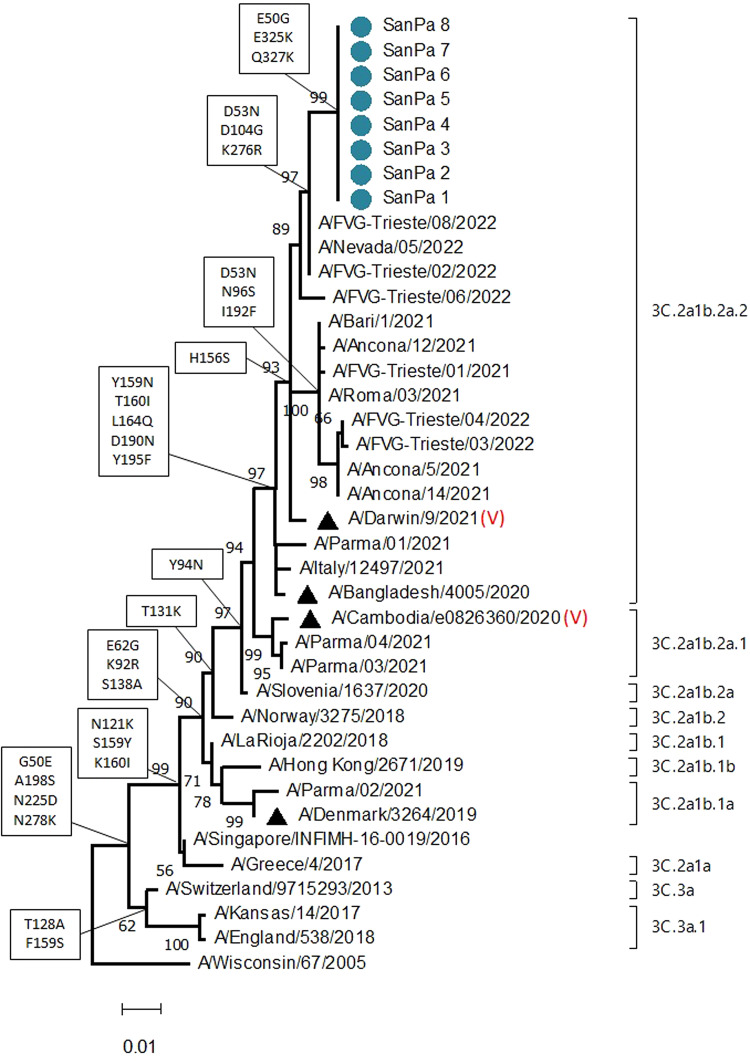
Phylogenetic tree of partial HA gene (882 nt) of influenza A(H3N2) constructed with the maximum-likelihood method, based on the Hasegawa-Kishono-Yano (HKY) + G model [5] identified as the best-fitting model after the model test analysis, using MEGA 11 [6]. A discrete Gamma distribution was used to model evolutionary rate differences among sites (5 categories (+G = 0.1910)). Branch support (1000 bootstrap iterations) is provided next to nodes. Clades are indicated by square brackets. Phylogenetic analysis was conducted using reference sequences and Italian sequences available in GISAID with collection dates in the 2021/2022 influenza season. Sequences involved in the San Patrignano community outbreak are indicated by a light blue circle, reference strains used to classify subclades are labelled with black triangles. Vaccine strains are indicated by (V). Amino acid mutations associated with main nodes are indicated in squared boxes.

Nasopharyngeal swabs collected from eight individuals with influenza-like symptoms during the peak and the end of the epidemic tested positive for A(H3N2) influenza by real time RT-PCR using a commercial kit (Clonit Srl, Italy). Molecular characterisation was conducted for all eight strains by sequencing the genetic fragment coding for the HA1 subunit of the haemagglutinin (HA) protein (882 nt), as previously described [3]. Phylogenetic analysis was conducted using reference sequences and Italian sequences recovered from the GISAID EpiFlu database [4] with collection dates in the 2021/2022 influenza season. The phylogenetic tree demonstrated that San Patrignano community sequences were 100% identical to each other, confirming a common origin (Fig. 2). Additionally, no sequences identical to those obtained in this study were identified in public databases. All viruses belonged to the A(H3N2) Bangladesh-like (3C.2a1b.2a.2) clade, which is genetically distinct from the 2021/2022 influenza vaccine strain (A/Cambodia/e0826360/2020, 3C.2a1b.2a.1 clade).

## Discussion

The level of influenza activity during the 2021/2022 season across the WHO European Region was slightly higher than during the 2020/2021 season but still lower compared to pre-pandemic periods [7]. The reduced number of detected cases could be related to the measures introduced to slow the spread of SARS-CoV-2. However, as preventive measures gradually became less stringent, a very late increase in seasonal influenza transmission has been observed and new influenza outbreaks have been reported [8, 9].

During the 2021/2022 influenza season, A(H3N2) viruses have been dominant worldwide, with the predominance of strains from the clade 3C.2a1b.2a.2. For this reason, the WHO recommended to change the A(H3N2) vaccine component for the 2022/2023 influenza season to include a 3C.2a1b.2a.2 virus [10].

We report a large outbreak of A(H3N2) influenza that occurred in a residential rehabilitation community for drug users. This was the first influenza outbreak observed in the community since the beginning of the COVID-19 pandemic, and notably it occurred late in the influenza season, like in the rest of Italy.

Importantly, outbreak-related sequences did not belong to the 2021/2022 vaccine clade but fell into the same clade (3C.2a1b.2a.2) as the 2022/2023 season vaccine component (A/Darwin/9/2021) [10]. A mismatch between the circulating strain and the vaccine strains used for immunisations in the community may explain the lack of protection observed in our study. However, a multivariable analysis to confirm that the vaccine effectiveness was low was not performed. Reduced antibody titres 5 months after vaccinations could have also contributed to the lack of protection in this outbreak.

Compared to the COVID-19 outbreak that occurred between October and December 2020 (before the vaccination programme started), which had an attack rate as high as 65%, symptoms were considerably more severe but with a shorter duration (data not shown).

Our results showed a higher attack rate among residents who worked and slept in close proximity.

The catering and food service, which was the most affected working area, was also the one with the most interaction with other areas. Notably, the VE estimated in catering workers – the highest risk group in our study – suggested that the vaccine was somewhat effective in this group.

San Patrignano is not a confined community, since residents have interactions with outsiders. Although phylogenetic analysis was performed on a small number of cases, sequences collected at the peak and during the last phase of the epidemic were identical to each other, suggesting that one single A(H3N2) strain was involved in the outbreak.

The attack rate (26.2%) was higher than the one observed in another influenza outbreak that occurred within the same community during the 2003/2004 season (15.9%), which was caused by a drifted A(H3N2) virus [11]. The high attack rate could be attributed to the intrinsic characteristics of the Bangladesh-like lineage and to the absence of natural boosters as a consequence of no influenza virus circulation during the previous season.

Despite the SARS-CoV-2 emergency pushed influenza viruses into the background, they remain an important public health threat. Especially in community settings, where implementing social distancing measures and quarantine is challenging and close and multiple contacts may facilitate transmission, vaccination represents the main prevention strategy, although the continuous emergence of drifted strains makes surveillance and vaccine formulation updates crucial for infection control.

## Data Availability

The data presented in this study are available on request from the corresponding author. Sequence data that support the findings of this study have been deposited in GenBank with the accession numbers ON965372–ON965379.

## Supplementary material

For supplementary material accompanying this paper visit http://doi.org/10.1017/S0950268823000055. click here to view supplementary material
