# Supplementary material for: A large A(H3N2) influenza outbreak with a high attack rate in a drug user community in Italy, April 2022
Source: Epidemiol Infect. 2023 Jan 19;151:e36. doi: 10.1017/S0950268823000055 (PMC9990381; doi:10.1017/S0950268823000055)
Supplement: Supplementary file 1 [file S0950268823000055sup001.docx]

**Table S1** Attack rate, incidence, relative risk, and vaccine effectiveness among working areas in San Patrignano.

| **Working area** | **Total residents (N)** | **Cases** | **Attack rate (%)** | **Vaccinated** | **Non vaccinated** | **Cases among vaccinated cases** | **Cases among non-vaccinated cases** | **Incidence among vaccinated cases** | **Incidence among non-vaccinated cases** | **Relative Risk** | **Vaccine Effectiveness** |
| --- | --- | --- | --- | --- | --- | --- | --- | --- | --- | --- | --- |
| Animal farm | 39 | 0 | 0 | 11 | 28 | 0 | 0 | 0,00 | 0,00 | 0,00 | 1,00 |
| Bakery | 72 | 5 | 6.9 | 36 | 36 | 3 | 2 | 8,33 | 5,56 | 1,50 | -0,50 |
| Blacksmith's workshop | 12 | 3 | 25.0 | 1 | 11 | 0 | 3 | 0,00 | 27,27 | 0,00 | 1,00 |
| Building | 6 | 0 | 0 | 1 | 5 | 0 | 0 | 0,00 | 0,00 | 0,00 | 1,00 |
| Butchery | 23 | 8 | 34.8 | 20 | 3 | 7 | 1 | 35,00 | 33,33 | 1,05 | -0,05 |
| Catering and food service | 39 | 19 | 48.7 | 26 | 13 | 11 | 8 | 42,31 | 61,54 | 0,69 | 0,31 |
| Cheese factory | 57 | 12 | 21.1 | 19 | 38 | 6 | 6 | 31,58 | 15,79 | 2,00 | -1,00 |
| Children's centre (females) | 15 | 4 | 26.7 | 0 | 15 | 0 | 4 | 0,00 | 26,67 | 0,00 | 1,00 |
| Children's centre (males) | 11 | 3 | 27.3 | 0 | 11 | 0 | 3 | 0,00 | 27,27 | 0,00 | 1,00 |
| Decoration | 36 | 8 | 22.2 | 10 | 26 | 3 | 5 | 30,00 | 19,23 | 1,56 | -0,56 |
| Plumbing and electrical maintenance | 31 | 7 | 22.6 | 11 | 20 | 3 | 4 | 27,27 | 20,00 | 1,36 | -0,36 |
| Farm | 65 | 25 | 38.5 | 52 | 13 | 17 | 8 | 32,69 | 61,54 | 0,53 | 0,47 |
| Graphic design | 32 | 11 | 34.4 | 22 | 10 | 10 | 1 | 45,45 | 10,00 | 4,55 | -3,55 |
| Kennel | 22 | 3 | 13.6 | 8 | 14 | 1 | 2 | 12,50 | 14,29 | 0,88 | 0,13 |
| Kitchen | 46 | 17 | 37.0 | 29 | 17 | 10 | 7 | 34,48 | 41,18 | 0,84 | 0,16 |
| Laundry | 25 | 10 | 40.0 | 14 | 11 | 7 | 3 | 50,00 | 27,27 | 1,83 | -0,83 |
| Medical centre | 23 | 8 | 34.8 | 18 | 5 | 8 | 0 | 44,44 | 0,00 | 0,00 | 1,00 |
| Park | 74 | 21 | 28.4 | 33 | 41 | 9 | 12 | 27,27 | 29,27 | 0,93 | 0,07 |
| Pizzeria | 1 | 0 | 0 | 0 | 1 | 0 | 0 | 0,00 | 0,00 | 0,00 | 1,00 |
| Vineyard | 5 | 0 | 0 | 4 | 1 | 0 | 0 | 0,00 | 0,00 | 0,00 | 1,00 |
| Warehouse | 32 | 12 | 37.5 | 22 | 10 | 6 | 6 | 27,27 | 60,00 | 0,45 | 0,55 |
| Weaving | 36 | 8 | 22.2 | 3 | 33 | 2 | 6 | 66,67 | 18,18 | 3,67 | -2,67 |
| Total | 702 | 184 |  | 340 | 362 | 103 | 81 | 30,3 | 22,4 | 1,35 | -0,35 |
